# Association of different serum creatinine trajectories with 28-day mortality in patients with acute kidney injury on chronic kidney disease: based on the MIMIC-IV database

**DOI:** 10.1186/s40001-025-03632-x

**Published:** 2025-12-15

**Authors:** Jun Ying, Hanjing Zhou, Yingxin Zhang, Shan Zhu, Gancong Zhang, Jian Huang

**Affiliations:** 1 Department of Nephrology, Affiliated Jinhua Hospital, Zhejiang University School of Medicine, Jinhua, 321000 China; 2 Central Health Center of Caozhai Town, Jindong District, Jinhua, 321000 China

**Keywords:** Serum creatinine, 28-day mortality, Trajectory analysis, Chronic kidney disease, Acute kidney injury

## Abstract

**Background:**

To evaluate the relationship between short-term longitudinal serum creatinine (Scr) trajectories and 28-day mortality in patients with acute kidney injury (AKI) on chronic kidney disease (CKD).

**Methods:**

The data sources of this study were the Medical Information Mart for Intensive Care IV (MIMIC-IV) database. Group-Based Trajectory Modeling was used to classify the trajectories of Scr indices within 96 h after Intensive Care Unit admission. Kaplan–Meier survival curves were used to analyze the 28-day survival probabilities of patients with different Scr trajectories. Multivariate Cox proportional hazards models were applied to explore the association between different Scr trajectories and 28-day mortality. Receiver operating characteristic curves were employed to assess the predictive performance of the predictive model (model 3) for 28-day mortality. Decision curve analysis (DCA) was conducted to explore the clinical net benefit of the predictive model (model 3). Subgroup analysis was conducted to explore the robustness of the relationship.

**Results:**

A total of 7,852 patients with AKI on CKD were included in this study. Through GBTM analysis, five distinct trajectory groups were identified: Group G1 (*n* = 1762, 22.4%) with Scr levels maintained at approximately 1 and showing a stable trend (Scr low-stable group); Group G2 (*n* = 915, 11.7%) with baseline Scr levels above 8 and showing a decreasing trend (Scr high-decreasing group); Group G3 (*n* = 2017, 25.7%) with Scr levels maintained at approximately 1.3–1.5 and showing a stable trend (Scr moderate-low stable group); Group G4 (*n* = 1707, 21.7%) with Scr levels maintained at approximately 2 and showing a stable trend (Scr moderate-stable group); and Group G5 (*n* = 1451, 18.5%) with Scr levels maintained at approximately 4 and showing a stable trend (Scr moderate-high stable group). The 28-day survival probabilities in Groups G1, G2, and G3 were higher than that in Groups G4 and G5. Compared with Group G1, the risk of 28-day mortality increased by 0.819-fold in Group G2, by 0.454-fold in Group G4, and by 0.860-fold in Group G5. The area under the curve (AUC) of model 3 was 0.795 (95% CI 0.782–0.807). DCA results showed that when the threshold probability ranged from 5 to 85%, the net benefit of model 3 was significantly higher than that of the "treat all" and "treat none" models. Group G4 and Group G5 were associated with increased 28-day mortality in both the male and female subgroups.

**Conclusions:**

The results showed that AKI on CKD patients with Scr high-decreasing trend, Scr moderate-stable trend, and Scr moderate-high stable trend had a higher risk of 28-day mortality.

**Supplementary Information:**

The online version contains supplementary material available at 10.1186/s40001-025-03632-x.

## Introduction

Chronic kidney disease (CKD) is defined as a group of diseases characterized by structural or functional abnormalities of the kidneys lasting for more than 3 months [1]. In 2021, there were 19,935,038 new cases of CKD globally, with 673,722,703 prevalent cases and 1,527,629 deaths [2]. CKD is a major cause of chronic renal failure, cardiovascular diseases, and other conditions, posing a significant public health problem [3]. Acute kidney injury (AKI) is defined as a rapid increase in serum creatinine (Scr) levels, decreased urine output, or both [4]. AKI is a major risk factor for the development of CKD and end-stage renal disease [5]. Studies have found that 24.6% of AKI patients develop CKD within a 3-year follow-up period [6]. Conversely, CKD is also a risk factor for AKI [7]. CKD patients often develop AKI during hospitalization, significantly increasing short-term mortality risk. Given the substantial public health burden of both CKD and AKI, as well as the significant harm of AKI on CKD, urgent attention should be paid to AKI on CKD.

Scr, as a biomarker reflecting glomerular filtration function, has been widely used to assess renal function and diagnose kidney-related diseases [8]. The latest evidence showed that fluctuations in Scr are closely associated with the risk of death in CKD, but systematic trajectory typing research is lacking [9]. Trajectory studies can more strongly infer causal relationships through the sequence of time series. Group-based Trajectory Modeling (GBTM) is a common research method for time series. It can classify continuously measured data into several subgroups with similar evolutionary patterns [10]. Therefore, this study uses the Medical Information Mart for Intensive Care IV (MIMIC-IV) database to explore the association between Scr trajectories and 28-day mortality in AKI on CKD patients, which is of great significance for the early and precise identification of high-risk populations, as well as clinical intervention and resource allocation.

## Methods

### Data source

This study was a retrospective study. The data sources of this study was the MIMIC-IV databases. The MIMIC-IV database is a large publicly available intensive care dataset containing de-identified health-related data of Intensive Care Unit (ICU) patients from Beth Israel Deaconess Medical Center in the United States.

### Patient selection

**Fig. 1:**
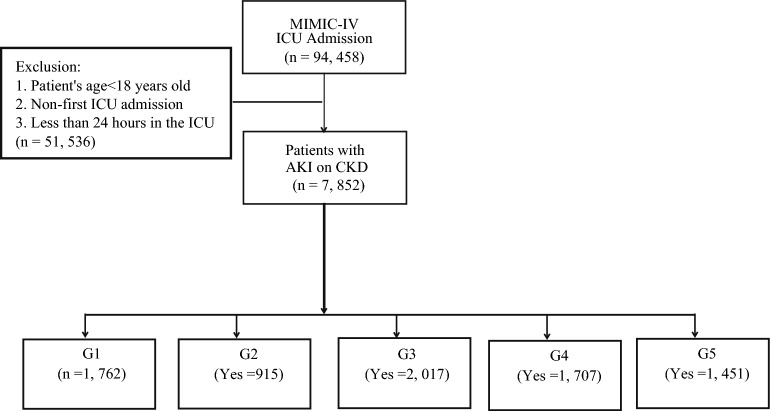
The flowchart of participants in the MIMIC-IV database in this study. *MIMIV-IV* Medical Information Mart for Intensive Care IV

Inclusion criteria: 1. patients with a pre-admission or discharge diagnosis of CKD according to the International Classification of Diseases (ICD): ICD-9 code (585.x) or ICD-10 code (N18.x); 2. patients who develop AKI during hospitalization were diagnosed according to the Kidney Disease: Improving Global Outcomes (KDIGO) criteria: an increase in Scr of ≥ 0.3 mg/dL within 48 h, or an increase in Scr of ≥ 50% from the baseline within 7 days. Exclusion criteria: 1. patients aged < 18 years; 2. patients were not first-time ICU admissions; 3. patients with an ICU length of stay < 24 h. The patient screening processes for the MIMIC-IV were shown in Fig. 1.

### Outcome measure

The outcome of this study was 28-day mortality.

### Data collection

This study collected age, gender (female, male), marital status (divorced, married, single, widowed), race (black, other, white), weight, diabetes (no, yes), congestive heart failure (no, yes), chronic pulmonary disease (no, yes), cerebrovascular disease (no, yes), rheumatic disease (no, yes), sepsis (no, yes), white blood cell (WBC), blood urea nitrogen (BUN), anion gap, total calcium, glucose, potassium, sodium, bicarbonate, chloride, Scr-baseline, Physiology Score II (SAPSII), Charlson Comorbidity Index (CCI), Sequential Organ Failure Assessment (SOFA), continuous renal replacement therapy (CRRT), renal replacement therapy (RRT), proteinuria, output urine, estimated glomerular filtration rate (eGFR), Δ eGFR, Δ eGFR percentage and hospital length of stay. For variables with missing data ≤ 20%, multiple imputation was performed, while those with missing data > 20% were excluded.

### Serum creatinine measurement

Scr was measured and recorded at least three times during the first 96 h after admission to the ICU. In the case of multiple recordings of Scr in 6-h intervals, we calculated the mean of these recordings for further analysis in the present study. The Scr missing data were handled consistently with other covariates: for variables with missing data ≤ 20%, multiple imputation was performed, while those with missing data > 20% were excluded.

### Statistical analysis

R 4.4.3 software was used for data description and statistical analysis. This study employed GBTM to analyze the trajectories of Scr indices within 96 h after ICU admission. To ensure an adequate sample size in each group, we constructed models with 2 to 5 groups and assigned group memberships accordingly. The optimal number of groups was determined by evaluating the following parameters: lower absolute values of Bayesian Information Criterion (BIC), Akaike Information Criterion (AIC), Consistent Akaike Information Criterion (CAIC), and Hannan–Quinn Information Criterion (HQIC); and an average posterior probability (AvePP) > 0.7 for each group. After determining the trajectories, normally distributed quantitative data were presented as mean [standard deviation (SD)] and analyzed using Student’s t-test. Non-normally distributed quantitative data were expressed as median (interquartile range [IQR]) and analyzed using the Mann–Whitney U test. Categorical variables were described as numbers (percentages) and analyzed using the Chi-square test. Kaplan–Meier (KM) survival curves were used to analyze the 28-day survival probabilities of patients with different Scr trajectories. Multivariate Cox proportional hazards models were used to explore the relationship between different Scr trajectories and 28-day mortality across three models (Model 1, Model 2, and Model 3). The results are presented as hazard ratios (HR) with 95% confidence intervals (95% CI). Model 1 included no adjustments. Model 2 was adjusted for age, gender, race, marital status, and weight. Model 3 was adjusted for age, gender, marital status, race, weight, diabetes, congestive heart failure, chronic pulmonary disease, cerebrovascular disease, rheumatic disease, sepsis, WBC, BUN, anion gap, total calcium, glucose, potassium, sodium, bicarbonate, chloride, Scr-baseline, SAPSII, CCI, SOFA, CRRT, RRT, proteinuria, output urine, eGFR, Δ eGFR. Model 3 was identified as the predictive model in this study. Receiver operating characteristic (ROC) curves were used to evaluate the predictive performance of the predictive model, SOFA and SAPSII in predicting 28-day mortality. Decision curve analysis (DCA) was performed to explore differences in the clinical net benefit of the predictive model. Subgroup analysis was stratified by Scr-baseline and sex to explore the robustness of the relationship. Statistical significance was defined as *P* < 0.05.

## Results

### Serum creatinine trajectories

**Fig. 2:**
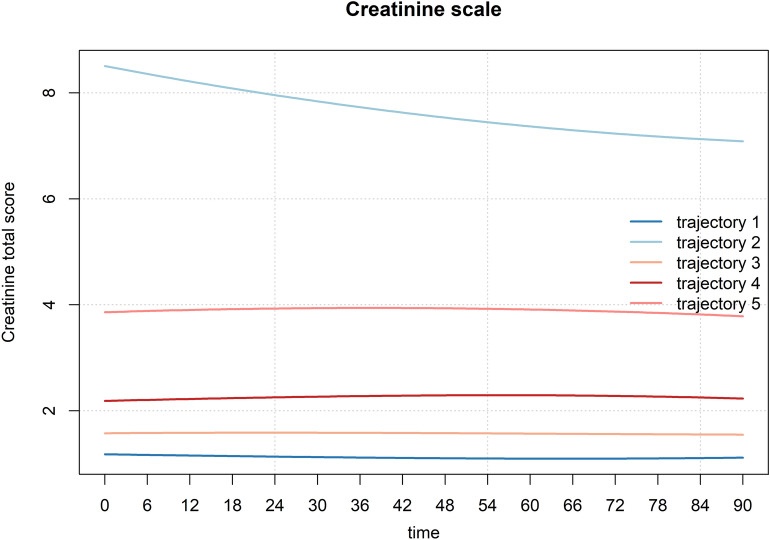
Trajectories of serum creatinine within 96 h after ICU admission in patients with CKD complicated by AKI. *ICU* Intensive Care Unit, *CKD* chronic kidney disease, *AKI* acute kidney injury

A total of 7,852 patients with AKI on CKD were included in this study. GBTM analysis identified 5 distinct trajectories (Fig. 2 and Table 1). These groups exhibited high AvePP (> 0.97) and the smallest BIC, AIC, and HQIC values. The five trajectory groups were as follows: Group G1 (*n* = 1762, 22.4%) with Scr levels maintained at approximately 1 and showing a stable trend (Scr low-stable group); Group G2 (*n* = 915, 11.7%) with baseline Scr levels above 8 and showing a decreasing trend (Scr high-decreasing group); Group G3 (*n* = 2017, 25.7%) with Scr levels maintained at approximately 1.3–1.5 and showing a stable trend (Scr moderate-low stable group); Group G4 (*n* = 1707, 21.7%) with Scr levels maintained at approximately 2 and showing a stable trend (Scr moderate-stable group); and Group G5 (*n* = 1451, 18.5%) with Scr levels maintained at approximately 4 and showing a stable trend (Scr moderate-high stable group).

**Table 1:** Selection of trajectory groups

Model	AvePP	AIC	BIC	CAIC	SSBIC	HQIC
Trajectory 1	100%	569031.1	569060.3	569063.3	569050.7	569039.8
Trajectory 2	99.7%/99.7%	346578.1	346646.3	346653.3	346624.0	346598.6
Trajectory 3	99.3%/099.3%/98.7%	284827.2	284983.1	284999.1	284932.2	284874.0
Trajectory 4	99.0%/99.1%/98.2%/98.7%	248462.1	248666.6	248687.6	248599.9	248523.5
Trajectory 5	97.8%/98.9%/97.2%98.0%/98.6%	229370.9	229633.9	229660.9	229548.1	229449.9

*AvePP* average posterior probability, *AIC* Akaike Information Criterion, *BIC* Bayesian Information Criterion, *CAIC* Consistent Akaike Information Criterion, *SSBIC* Sample-Size Adjusted Bayesian Information Criterion, *HQIC* Hannan–Quinn Information Criterion

### Baseline characteristics of patients

The basic characteristics of AKI on CKD patients across different trajectory groups are shown in Table 2. Compared with other groups, Group G1 (76.32 years, SD = 11.81) and Group G3 (76.02 years, SD = 11.53) had higher mean ages. Group G1 also had a higher proportion of females (*N* = 781, 44.3%). In females, Group G1 (27.1%) and Group G3 (24.1%) were more represented, whereas in males, Group G3 (26.6%) and Group G4 (22.3%) accounted for a higher proportion (Supplementary Table 1). There were differences in marital status and race distribution among the groups. In terms of comorbidities, Group G5 had a significantly higher prevalence of diabetes and sepsis; Group G4 had a higher prevalence of chronic pulmonary disease and congestive heart failure; and Group G1 had a higher prevalence of cerebrovascular diseases. Regarding prognostic scores, Group G1 had lower SAPS II and SOFA scores, while Group G2 had a lower CCI score. Significant differences were observed among the five groups in WBC, BUN, anion gap, total calcium, glucose, potassium, sodium, bicarbonate, and chloride levels. Group G2 had the highest Scr-baseline levels and Δ eGFR percentage. Group G2 and Group G5 had a higher demand for CRRT and RRT. Group G1 showed the highest urine output, eGFR levels, and Δ eGFR. There were also differences in 28-day mortality among the five groups, with Groups G4 and G5 having higher 28-day mortality rates.

**Table 2:** Baseline characteristics of patients

Level	G1	G2	G3	G4	G5	*P*
N	1762	915	2017	1707	1451	
Gender (%)						
Female	781 (44.3)	280 (30.6)	694 (34.4)	600 (35.1)	527 (36.3)	< 0.001
Male	981 (55.7)	635 (69.4)	1323 (65.6)	1107 (64.9)	924 (63.7)	
Age, mean (SD)	76.32 (11.81)	62.62 (14.47)	76.02 (11.53)	74.26 (12.52)	71.53 (12.87)	< 0.001
Marital status (%)						
Divorced	135 (7.7)	72 (7.9)	165 (8.2)	103 (6.0)	123 (8.5)	< 0.001
Married	851 (48.3)	436 (47.7)	1011 (50.1)	889 (52.1)	736 (50.7)	
Single	399 (22.6)	333 (36.4)	402 (19.9)	378 (22.1)	370 (25.5)	
Widowed	377 (21.4)	74 (8.1)	439 (21.8)	337 (19.7)	222 (15.3)	
Race (%)						
Black	180 (10.2)	250 (27.3)	227 (11.3)	208 (12.2)	210 (14.5)	< 0.001
Other	354 (20.1)	253 (27.7)	392 (19.4)	372 (21.8)	337 (23.2)	
White	1228 (69.7)	412 (45.0)	1398 (69.3)	1127 (66.0)	904 (62.3)	
Hospital length of stay, mean (SD)	12.27 (10.97)	13.79 (13.69)	12.64 (11.68)	14.15 (13.59)	13.95 (16.86)	< 0.001
28-day mortality (%)						
No	1519 (86.2)	777 (84.9)	1697 (84.1)	1266 (74.2)	1034 (71.3)	< 0.001
Yes	243 (13.8)	138 (15.1)	320 (15.9)	441 (25.8)	417 (28.7)	
Weight, mean (SD)	81.61 (22.47)	84.38 (24.10)	85.47 (23.54)	85.44 (24.60)	83.69 (24.52)	< 0.001
Diabetes (%)						
No	1007 (57.2)	398 (43.5)	1007 (49.9)	840 (49.2)	624 (43.0)	< 0.001
Yes	755 (42.8)	517 (56.5)	1010 (50.1)	867 (50.8)	827 (57.0)	
Congestive heart failure (%)						
No	996 (56.5)	491 (53.7)	927 (46.0)	669 (39.2)	636 (43.8)	< 0.001
Yes	766 (43.5)	424 (46.3)	1090 (54.0)	1038 (60.8)	815 (56.2)	
Chronic pulmonary disease (%)						
No	1240 (70.4)	736 (80.4)	1432 (71.0)	1176 (68.9)	1033 (71.2)	< 0.001
Yes	522 (29.6)	179 (19.6)	585 (29.0)	531 (31.1)	418 (28.8)	
Cerebrovascular disease (%)						
No	1403 (79.6)	793 (86.7)	1661 (82.4)	1442 (84.5)	1234 (85.0)	< 0.001
Yes	359 (20.4)	122 (13.3)	356 (17.6)	265 (15.5)	217 (15.0)	
Rheumatic disease (%)						
No	1679 (95.3)	878 (96.0)	1936 (96.0)	1625 (95.2)	1397 (96.3)	0.478
Yes	83 (4.7)	37 (4.0)	81 (4.0)	82 (4.8)	54 (3.7)	
SAPSII, mean (SD)	39.58 (11.65)	44.62 (13.79)	42.12 (12.18)	46.21 (13.51)	48.12 (14.10)	< 0.001
SOFA, mean (SD)	5.49 (3.13)	8.43 (3.24)	6.18 (3.25)	7.86 (3.73)	8.81 (3.75)	< 0.001
CCI, mean (SD)	8.10 (2.32)	6.78 (2.61)	8.26 (2.28)	8.28 (2.24)	8.14 (2.45)	< 0.001
WBC, mean (SD)	10.81 (8.20)	10.62 (6.59)	10.75 (7.65)	12.27 (12.53)	11.85 (7.39)	< 0.001
BUN, mean (SD)	25.38 (12.26)	68.62 (35.17)	34.63 (16.21)	47.18 (23.46)	60.25 (31.87)	< 0.001
Anion gap, mean (SD)	13.86 (3.70)	20.12 (5.03)	14.56 (3.57)	15.68 (4.14)	17.65 (4.77)	< 0.001
Total calcium, mean (SD)	8.62 (0.78)	8.56 (1.12)	8.67 (0.83)	8.58 (0.90)	8.46 (0.96)	< 0.001
Chloride, mean (SD)	103.01 (5.97)	97.97 (7.04)	103.08 (6.09)	102.98 (7.08)	101.52 (7.45)	< 0.001
Glucose, mean (SD)	145.36 (86.86)	151.51 (103.92)	154.69 (82.00)	161.96 (93.91)	158.65 (100.72)	< 0.001
Potassium, mean (SD)	4.15 (0.65)	4.85 (0.99)	4.33 (0.73)	4.46 (0.83)	4.64 (0.92)	< 0.001
Sodium, mean (SD)	139.00 (28.99)	136.90 (5.37)	138.30 (5.00)	137.88 (5.70)	137.25 (5.66)	0.001
Bicarbonate, mean (SD)	23.91 (4.31)	21.73 (5.74)	23.42 (4.49)	22.10 (4.97)	21.02 (5.48)	< 0.001
Scr-baseline, mean (SD)	1.19 (0.29)	8.43 (3.61)	1.59 (0.37)	2.20 (0.68)	3.87 (1.38)	< 0.001
CRRT (%)						
No	1729 (98.1)	695 (76.0)	1939 (96.1)	1495 (87.6)	1113 (76.7)	< 0.001
Yes	33 (1.9)	220 (24.0)	78 (3.9)	212 (12.4)	338 (23.3)	
RRT (%)						
No	1718 (97.5)	182 (19.9)	1922 (95.3)	1448 (84.8)	771 (53.1)	< 0.001
Yes	44 (2.5)	733 (80.1)	95 (4.7)	259 (15.2)	680 (46.9)	
Proteinuria (%)						
No	1760 (99.9)	914 (99.9)	2016 (100.0)	1707 (100.0)	1451 (100.0)	0.466
Yes	2 (0.1)	1 (0.1)	1 (0.0)	0 (0.0)	0 (0.0)	
Output urine, mean (SD)	216.18 (210.66)	127.76 (206.41)	203.21 (223.03)	182.45 (202.41)	150.75 (192.79)	< 0.001
eGFR, mean (SD)	59.39 (18.41)	7.49 (4.26)	43.86 (14.11)	31.90 (13.70)	17.47 (10.15)	< 0.001
Sepsis (%)						
No	835 (47.4)	366 (40.0)	933 (46.3)	594 (34.8)	443 (30.5)	< 0.001
Yes	927 (52.6)	549 (60.0)	1084 (53.7)	1113 (65.2)	1008 (69.5)	
Δ eGFR, mean (SD)	4.59 (21.24)	2.14 (8.76)	1.14 (18.79)	−0.96 (20.12)	0.22 (14.65)	< 0.001
Δ eGFR percentage, mean (SD)	14.64 (41.62)	56.49 (158.78)	12.05 (49.54)	16.51 (83.21)	29.13 (109.77)	< 0.001

*WBC* white blood cell, *BUN* blood urea nitrogen, *SAPSII* Simplified Acute Physiology Score II, *CCI* Charlson Comorbidity Index, *SOFA* Sequential Organ Failure Assessment, *Scr* serum creatinine, *CRRT* continuous renal replacement therapy, *RRT* renal replacement therapy, *eGFR* estimated glomerular filtration rate

### 28-day survival curves for different serum creatinine trajectory groups

**Fig. 3:**
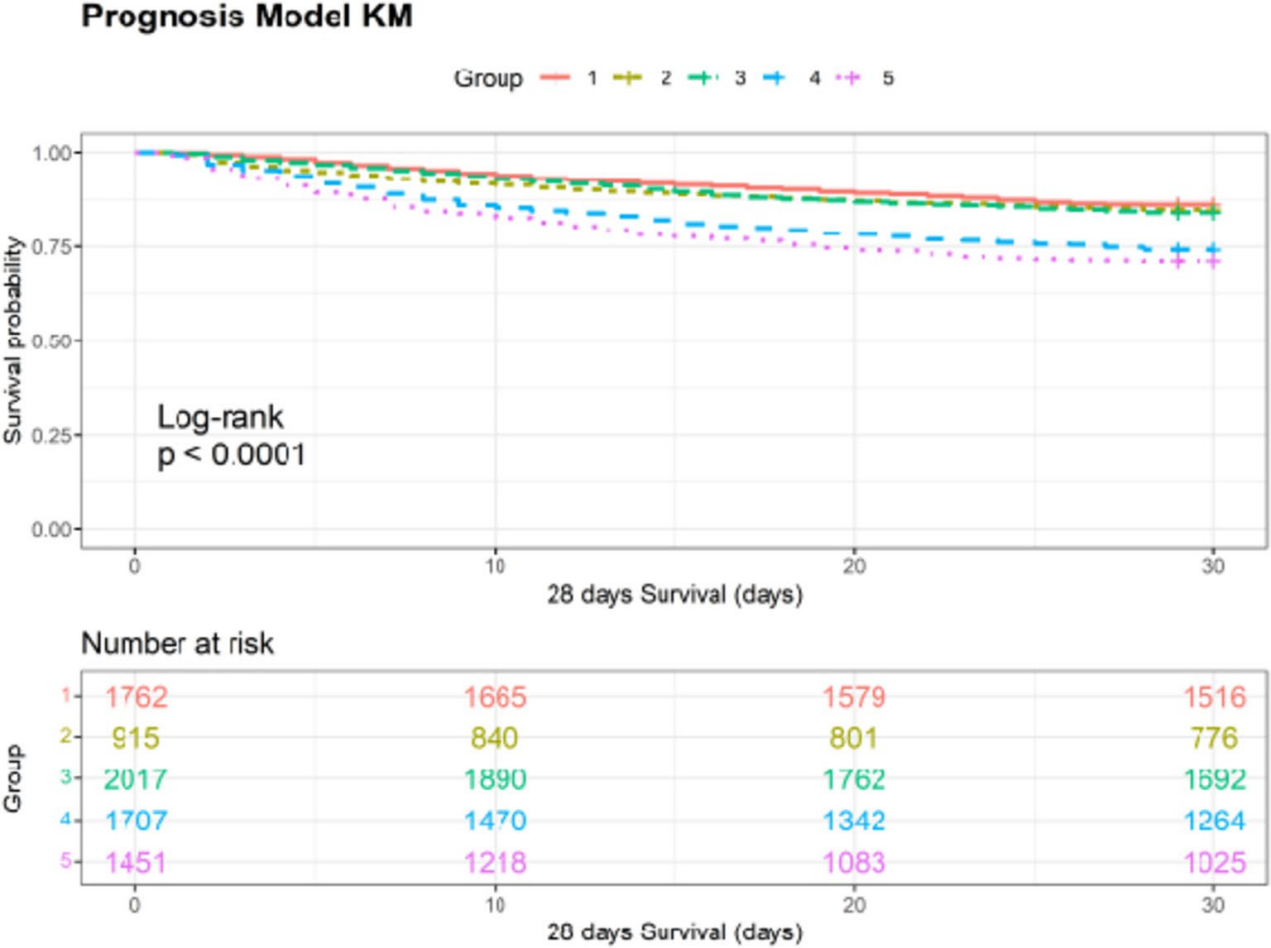
28-day survival curves for different serum creatinine trajectory groups

As shown in Fig. 3, there were differences in 28-day mortality among the five Scr trajectory groups. The 28-day survival probabilities in Groups G1, G2, and G3 were higher than that in Groups G4 and G5. Group G5 had the highest 28-day mortality rate, while Group G1 had the lowest.

### Relationship between serum creatinine trajectory groups and 28-day mortality

In model 1, compared with Group G1, Group G4 (HR = 2.039, 95% CI: 1.743–2.384, *P* < 0.001) and Group G5 (HR = 2.349, 95% CI: 2.005–2.751, *P* < 0.001) showed a significantly increased risk of 28-day mortality (Table 3).

**Table 3:** Relationship between serum creatinine trajectory groups and 28-day mortality

Reference	Trajectory group	*P*	HR (95% CI)
Model 1	Group G1 (Reference)		
	Group G2	0.288	1.120 (0.909–1.380)
	Group G3	0.072	1.165 (0.986–1.377)
	Group G4	< 0.001	2.039 (1.743–2.384)
	Group G5	< 0.001	2.349 (2.005–2.751)
Model 2	Group G1 (Reference)		
	Group G2	< 0.001	1.699 (1.366–2.113)
	Group G3	0.021	1.217 (1.029–1.438)
	Group G4	< 0.001	2.229 (1.905–2.609)
	Group G5	< 0.001	2.783 (2.371–3.267)
Model 3	Group G1 (Reference)		
	Group G2	0.007	1.819 (1.176–2.816)
	Group G3	0.446	1.081 (0.885–1.319)
	Group G4	0.003	1.454 (1.139–1.856)
	Group G5	< 0.001	1.860 (1.362–2.540)

Model 1 without adjustments. Model 2 was adjusted for age, gender, race, marital status, and weight. Model 3 was adjusted for age, gender, marital status, race, weight, diabetes, congestive heart failure, chronic pulmonary disease, cerebrovascular disease, rheumatic disease, sepsis, WBC, BUN, anion gap, total calcium, glucose, potassium, sodium, bicarbonate, chloride, Scr-baseline, SAPSII, CCI, SOFA, CRRT, RRT, proteinuria, output urine, eGFR, Δ eGFR

In model 2, compared with Group G1, the risk of 28-day mortality was significantly increased in Group G2 (HR = 1.699, 95% CI: 1.366–2.113, *P* < 0.001), Group G3 (HR = 1.217, 95% CI: 1.029–1.438, *P* = 0.021), Group G4 (HR = 2.229, 95% CI: 1.905–2.609, *P* < 0.001), and Group G5 (HR = 2.783, 95% CI: 2.371–3.267, *P* < 0.001) (Table 3).

In model 3, compared with Group G1, the risk of 28-day mortality increased by 0.819-fold in Group G2 (HR = 1.819, 95% CI: 1.176–2.816, *P* = 0.007), by 0.454-fold in Group G4 (HR = 1.454, 95% CI: 1.139–1.856, *P* = 0.003) and by 0.860-fold in Group G5 (HR = 1.860, 95% CI: 1.362–2.540, *P* < 0.001) (Table 3).

### Predictive performance and clinical net benefit of predictive model

**Fig. 4:**
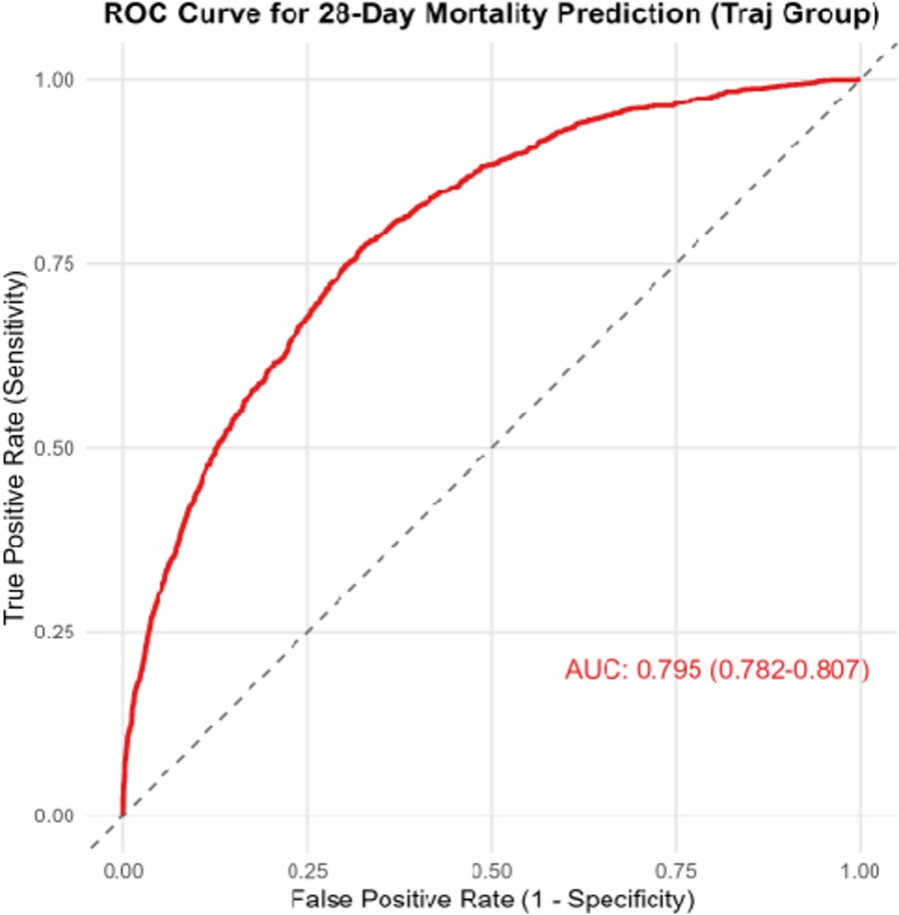
ROC curve of the predictive model for 28-day mortality in patients with CKD complicated by AKI. *ROC* receiver operating characteristic, *CKD* chronic kidney disease, *AKI* acute kidney injury

**Fig. 5:**
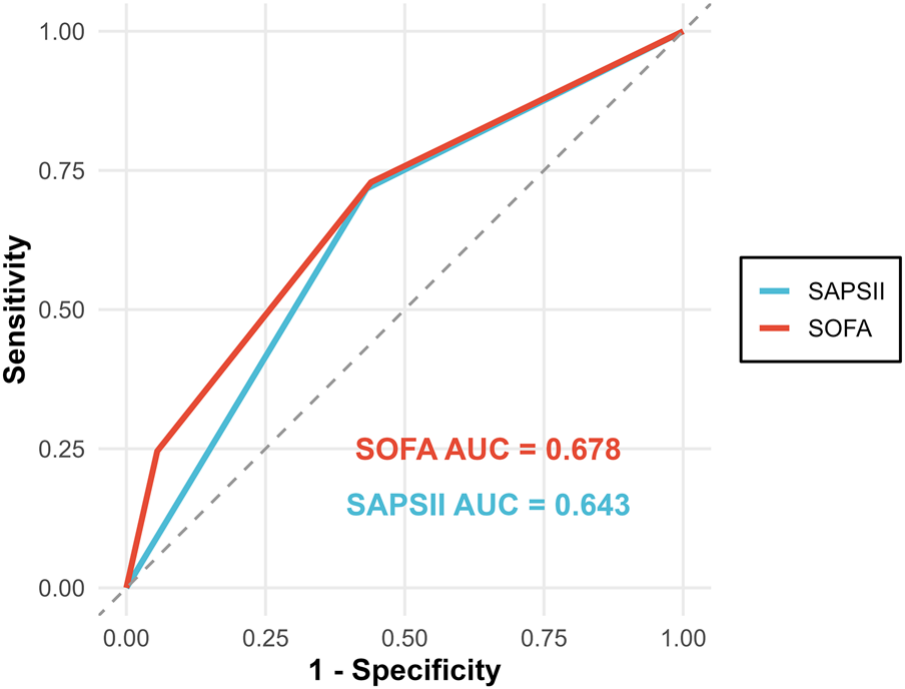
ROC curve of SOFA and SAPSII for 28-day mortality in patients with CKD complicated by AKI. *ROC* receiver operating characteristic, *SOFA* Sequential Organ Failure Assessment, *SAPSII* Simplified Acute Physiology Score II, *CKD* chronic kidney disease, *AKI* acute kidney injury

**Fig. 6:**
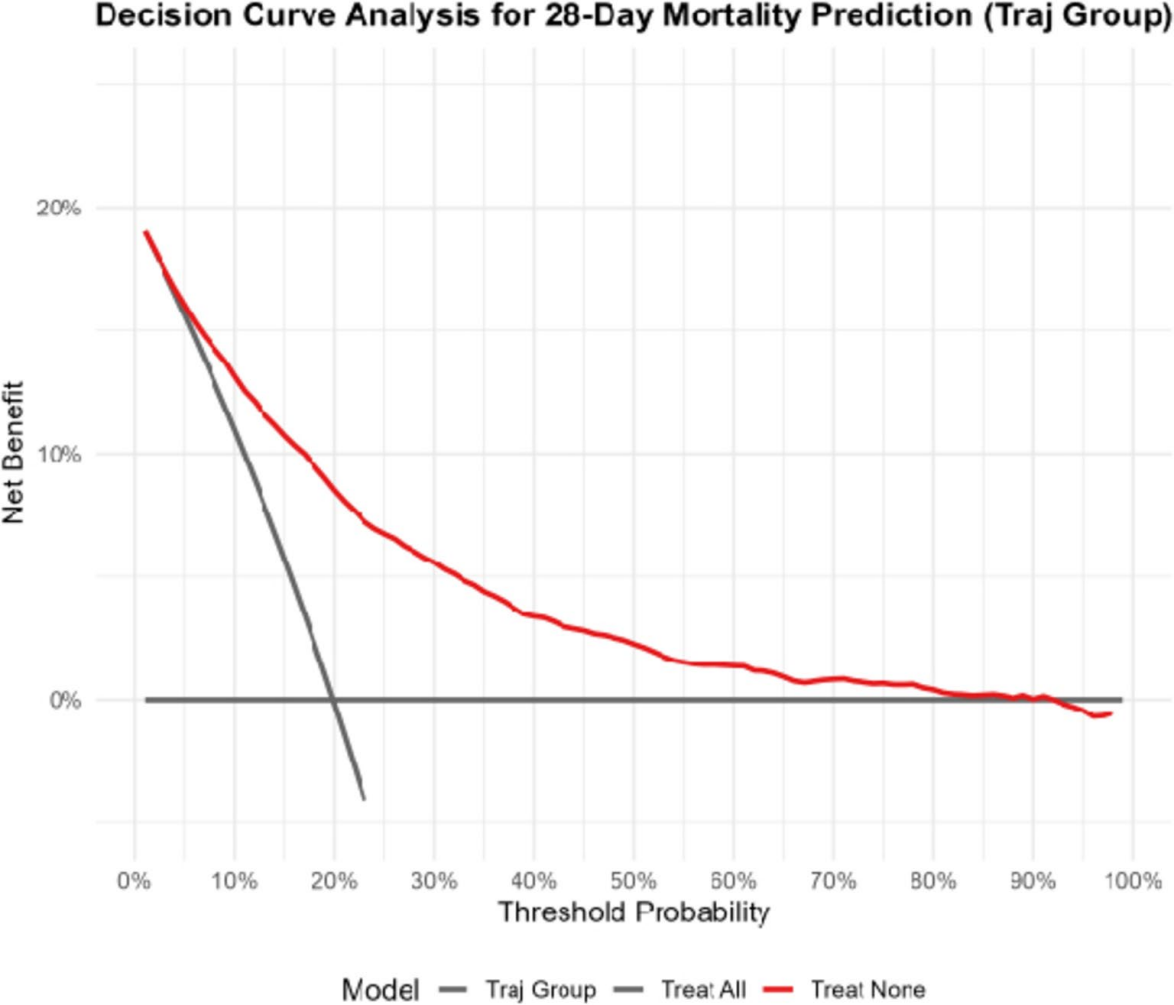
Clinical net benefit of the predictive model

The model 3 was identified as the predictive model in this study. ROC curves were constructed to explore its ability to predict 28-day mortality in patients with AKI on CKD. As shown in Fig. 4, the area under the curve (AUC) of the predictive model was 0.795 (95% CI 0.782–0.807). The AUCs of SOFA and SAPSII for predicting 28-day mortality in patients with AKI on CKD were 0.678 and 0.643, respectively (Fig. 5). The predictive performance of the predictive model was superior to that of SOFA and SAPSII. The DCA results indicated that when the threshold probability ranged from 5 to 85%, the net benefit of the predictive model was significantly higher than that of the "treat all" and "treat none" models (Fig. 6).

### Subgroup analysis

As shown in Table 4, Group G4 and Group G5 were associated with an increased 28-day mortality in both male and female subgroups. The Scr-baseline and Scr trajectory had an interaction, and the positive association of Group G4 and Group G5 with 28-day mortality was observed in the subgroup with Scr- baseline ≤ 1.8.

**Table 4:** Subgroup analysis

Subgroup	HR(95% CI)	*P*	*P* for interaction
Female			0.181
Group G1	Reference		
Group G2	0.95 (0.67–1.34)	0.756	
Group G3	1.09 (0.85–1.41)	0.499	
Group G4	1.72 (1.36–2.17)	< 0.001	
Group G5	1.46 (1.14–1.88)	0.003	
Male			
Group G1	Reference		
Group G2	0.83 (0.64–1.09)	0.176	
Group G3	0.92 (0.74–1.15)	0.465	
Group G4	1.35 (1.10–1.67)	0.005	
Group G5	1.59 (1.29–1.96)	< 0.001	
Scr-baseline (≤ 1.8)			0.042
Group G1	Reference		
Group G2	2.75 (0.68–11.06)	0.154	
Group G3	0.97 (0.81–1.16)	0.753	
Group G4	1.74 (1.41–2.14)	< 0.001	
Group G5	2.71 (1.86–3.96)	< 0.001	
Scr-baseline (> 1.8)			
Group G1	Reference		
Group G2	3.59 (0.50–25.64)	0.203	
GroupG3	4.13 (0.57–29.72)	0.159	
GroupG4	5.76 (0.81–41.03)	0.080	
Group G5	6.19 (0.87–44.09)	0.069	

*Scr* serum creatinine

## Discussion

Based on the MIMIC-IV database, this study explored the relationship between different Scr trajectory groups and 28-day mortality in patients with AKI on CKD. It was found that patients in Group G2 (Scr high-decreasing trend), Group G4 (Scr moderate-stable trend), and Group G5 (Scr moderate-high stable trend) had a higher risk of 28-day mortality.

In this study, distinct Scr trajectories were associated with varying 28-day mortality risks. In a study, AKI patients with a rapid increase in Scr had significantly higher 7-day mortality, and all-cause in-hospital mortality, with a positive correlation observed between Scr trajectory and these outcomes [11]. Scr trajectories have also been linked to prognosis in various other diseases. A retrospective cohort study categorized 7-day Scr trajectories into four groups and found that patients with a significant and persistent increase in Scr had significantly higher 1-year mortality after heart valve surgery compared to the other groups, and this trajectory was an independent risk factor for 1-year mortality [12]. Another retrospective cohort study on acute organophosphorus poisoning showed that compared to the "normal-consistent Scr" group, the "normal-increasing Scr" and "high-declining Scr" groups had higher ICU mortality, with HRs of 15.2 (95% CI: 4.2–54.6) and 15.7 (95% CI: 3.4–71.6), respectively [13]. A real-world study found that patients with increased Scr had a higher risk of in-hospital mortality, while those with normal baseline renal function and decreased Scr during hospitalization had a higher in-hospital mortality risk but a lower 90-day mortality risk [14]. In summary, the Scr trajectory can serve as an important indicator for assessing the short-term and long-term mortality risks of patients (especially critically ill or post-operative patients) and has significant reference value for clinical prognosis judgment.

The normal reference range for Scr is 0.7–1.4 mg/dL in males and 0.6–1.0 mg/dL in females [15]. In this study, the baseline Scr levels in Groups G2, G4, and G5 were significantly higher than these normal ranges. Elevated Scr reflects reduced glomerular filtration rate, which induces metabolic waste retention and water-electrolyte imbalance, increasing cardiovascular load and arrhythmia risk [16, 17]. In AKI on CKD, intra-renal inflammation is aggravated; pro-inflammatory factors trigger systemic inflammatory response syndrome to promote multi-organ dysfunction, while uremic toxins damage vascular endothelium, accelerating atherosclerosis and fatal cardiovascular events such as acute heart failure [18–21]. Additionally, impaired renal function causes abnormal protein metabolism and immune dysfunction, elevating infection risk and forming a vicious "infection–renal injury" cycle [22, 23]. Notably, Group G3 was not associated with increased 28-day mortality, which may be attributed to the lower Scr levels, lower SOFA scores, and higher eGFR levels in this group. The Scr levels in Group G3 remained stable at approximately 1.3–1.5, indicating that renal filtration function was essentially maintained to excrete metabolic wastes in the body. The lower SOFA scores suggest that the degree of multiple organ dysfunction in this group was mild, with a lower risk of systemic inflammatory response and organ failure, thereby reducing the likelihood of 28-day mortality due to multiple organ failure [24]. The higher eGFR levels indicate that the actual filtration capacity of the kidneys was adequate, further supporting the kidneys' compensatory capacity for metabolic demands and avoiding a series of life-threatening metabolic disorders caused by severe impairment of renal function [25].

The results of subgroup analysis showed that Group G4 and Group G5 were significantly associated with an increased risk of 28-day mortality in both male and female subgroups, suggesting that the adverse effects of these two trajectory groups on mortality have gender universality and are not interfered with by differences in physiology and clinical interventions caused by gender disparities. Particularly critical is that there was a significant interaction between Scr-baseline and Scr trajectory; the positive association of Group G4 and Group G5 with 28-day mortality was only observed in the subgroup with Scr -baseline ≤ 1.8. This may be because when Scr-baseline ≤ 1.8, the renal filtration function is fundamentally adequate, and abnormal changes in Scr trajectory at this time are more likely to reflect potential damage to renal function, thereby affecting prognosis. This finding provides a reference for clinical practice in formulating individualized prognostic assessment protocols for patients with different baseline Scr levels.

Traditional disease mortality risk assessment methods like prospective cohort studies usually categorize subjects by an indicator’s single-time-point measurement. However, such a single measurement is insufficient for mortality prediction, as it overlooks inter-individual heterogeneity and fails to reflect longitudinal data’s variation patterns and trends, thus limiting the description of inherent associations. Therefore, this study adopted trajectory analysis to explore the relationship between Scr and 28-day mortality in CKD patients complicated with AKI. Common trajectory analysis methods include GBTM, Growth Mixture Model (GMM), and cluster analysis. Relevant studies have shown that GMM performs best in single-trajectory analysis; GBTM has good overall performance and can be extended to multi-trajectory studies; cluster analysis works adequately for multi-trajectory analysis but requires high-quality follow-up data [26]. Thus, the choice of trajectory analysis method should be based on the specific characteristics of the data.

In this study, we identified distinct Scr trajectories and further analyzed their associations with 28-day mortality in AKI on CKD patients. Our findings may provide epidemiological evidence for understanding the changes in Scr levels after ICU admission and their correlation with 28-day mortality in this patient population. However, this study has several limitations. First, relying on a single-center dataset inherently limits generalizability. The MIMIC-IV database-derived cohort may not represent broader CKD or AKI populations, as patient characteristics, diagnostic criteria, and treatment protocols can vary significantly across medical institutions, regions, and healthcare systems. Future research must validate these results in independent, multi-center cohorts encompassing diverse patient populations—including different age groups, races, comorbidity spectrums, and varying levels of healthcare access. Second, we only analyzed the impact of Scr trajectories within 96 h of ICU admission on 28-day mortality; the effect of longer-term Scr trajectories on the prognosis of CKD patients with AKI requires further investigation.

## Conclusion

This study analyzed the impact of Scr trajectories on 28-day mortality in patients with AKI on CKD. The results showed that patients with a Scr high-decreasing trend, Scr moderate-stable trend, and Scr moderate-high stable trend, these three groups of patients, had a higher risk of 28-day mortality. These findings may help in the early identification of high-risk populations, facilitating clinical intervention and resource allocation.

## Supplementary Information

Supplementary material 1.

## Data Availability

The data sources of this study was the MIMIC-IV database.

## References

[1] Romagnani P, Agarwal R, Chan JCN, Levin A, Kalyesubula R, Karam S, et al. Chronic kidney disease. Nat Rev Dis Primers. 2025;11:8. 10.1038/s41572-024-00589-9. 39885176 10.1038/s41572-024-00589-9

[2] Guo J, Liu Z, Wang P, Wu H, Fan K, Jin J, et al. Global, regional, and national burden inequality of chronic kidney disease, 1990-2021: a systematic analysis for the global burden of disease study 2021. Front Med. 2024;11:1501175. 10.3389/fmed.2024.1501175. 10.3389/fmed.2024.1501175 PMC11774877 39882527

[3] Mayne KJ, Hanlon P, Lees JS. Detecting and managing the patient with chronic kidney disease in primary care: a review of the latest guidelines. Diabetes Obes Metab. 2024;26:43–54. 10.1111/dom.15625. 38699995 10.1111/dom.15625

[4] Ostermann M, Lumlertgul N, Jeong R, See E, Joannidis M, James M. Acute kidney injury. Lancet. 2025;405:241–56. 10.1016/s0140-6736(24)02385-7. 39826969 10.1016/S0140-6736(24)02385-7

[5] Yang X, Zhang T, Zhou H, Ni Z, Wang Q, Wu J, et al. Acute kidney injury as an independent predicting factor for stage 3 or higher chronic kidney disease after nephrectomy. Urol Oncol. 2023;41:149.e141-149.e149. 10.1016/j.urolonc.2022.10.011. 10.1016/j.urolonc.2022.10.011 36463084

[6] Yeh TH, Tu KC, Wang HY, Chen JY. From acute to chronic: unraveling the pathophysiological mechanisms of the progression from acute kidney injury to acute kidney disease to chronic kidney disease. Int J Mol Sci. 2024. 10.3390/ijms25031755. 38339031 10.3390/ijms25031755 PMC10855633

[7] Chang YH, Wu CH, Chou NK, Tseng LJ, Huang IP, Wang CH, et al. High plasma C-terminal FGF-23 levels predict poor outcomes in patients with chronic kidney disease superimposed with acute kidney injury. Ther Adv Chronic Dis. 2020;11:2040622320964161. 10.1177/2040622320964161. 33133477 10.1177/2040622320964161 PMC7576912

[8] He L, Yu J, Han G, Huang D, Han L, Zhang Q, et al. Analytical performance evaluation of different test systems on serum creatinine assay. J Clin Lab Anal. 2022;36:e24206. 10.1002/jcla.24206. 34957600 10.1002/jcla.24206 PMC8842161

[9] Efros O, Beckerman P, Basson AA, Cohen R, Klang E, Frenkel Nir Y, et al. Fluctuations in serum creatinine levels during hospitalization and long-term end-stage kidney disease and mortality. JAMA Netw Open. 2023;6:e2326996. 10.1001/jamanetworkopen.2023.26996. 37535358 10.1001/jamanetworkopen.2023.26996 PMC10401303

[10] Shen X, Li J, Yan H, Zhou S, Yang S, Li W. Combined blood pressure and heart rate trajectories are associated with prognosis in critically ill patients with acute aortic dissection: a group-based multi-trajectory analysis. Heliyon. 2024;10:e29934. 10.1016/j.heliyon.2024.e29934. 38707356 10.1016/j.heliyon.2024.e29934 PMC11066306

[11] Takkavatakarn K, Oh W, Chan L, Hofer I, Shawwa K, Kraft M, et al. Machine learning derived serum creatinine trajectories in acute kidney injury in critically ill patients with sepsis. Crit Care. 2024;28:156. 10.1186/s13054-024-04935-x. 38730421 10.1186/s13054-024-04935-x PMC11084026

[12] Cho JS, Choi M, Shim JK, Park JH, Shin HJ, Choi HW, et al. Association of serum creatinine trajectories with 1-year mortality after valvular heart surgery: a retrospective cohort study. Int J Surg. 2024;110:7097–105. 10.1097/js9.0000000000001933. 38990280 10.1097/JS9.0000000000001933 PMC11573049

[13] Farooqui WA, Uddin M, Qadeer R, Shafique K. Latent class trajectories of biochemical parameters and their relationship with risk of mortality in ICU among acute organophosphorus poisoning patients. Sci Rep. 2022;12:11633. 10.1038/s41598-022-15973-2. 35804092 10.1038/s41598-022-15973-2 PMC9270430

[14] Laszczyńska O, Severo M, Mascarenhas J, Paiva JA, Azevedo A. Serum creatinine trajectories in real-world hospitalized patients: clinical context and short-term mortality. J Invest Med. 2020;68:870–81. 10.1136/jim-2019-001185. 10.1136/jim-2019-001185 32179556

[15] Reganis MN, Stickle D. Effect of 1 vs. 2 decimal place reporting of creatinine (mg/dL) for calculation of estimated glomerular filtration rate (eGFR). Am J Clin Pathol. 2021;156:S30–1. 10.1093/ajcp/aqab191.059.

[16] Guzzo I, Picca S, Askenazi D. Neonatal kidney dysfunction. In: Schaefer F, Greenbaum LA, editors. Pediatric kidney disease. Cham: Springer International Publishing; 2023. p. 1437–68.

[17] Aziz N, Wal P, Sinha R, Shirode PR, Chakraborthy G, Sharma MC, et al. A comprehensive review on the significance of cysteine in various metabolic disorders; particularly CVD, diabetes, renal dysfunction, and ischemic stroke. Curr Protein Pept Sci. 2024;25:682–707. 10.2174/0113892037287215240424090908. 38766817 10.2174/0113892037287215240424090908

[18] Morelli MC, Rendina M, La Manna G, Alessandria C, Pasulo L, Lenci I, et al. Position paper on liver and kidney diseases from the Italian Association for the Study of Liver (AISF), in collaboration with the Italian Society of Nephrology (SIN). Dig Liver Dis. 2021;53:S49–86. 10.1016/j.dld.2021.03.035. 34074490 10.1016/j.dld.2021.03.035

[19] Raouf MM, Shadad EA, Ali NS. Effect of atorvastatin as a renal protective agent in patients with systemic inflammatory response syndrome using the renal arterial resistive index. Acute Crit Care. 2025;40:95–104. 10.4266/acc.003912. 39978955 10.4266/acc.003912 PMC11924345

[20] Harlacher E, Wollenhaupt J, Baaten CCFMJ, Noels H. Impact of uremic toxins on endothelial dysfunction in chronic kidney disease: a systematic review. Int J Mol Sci. 2022;23:531. 35008960 10.3390/ijms23010531 PMC8745705

[21] El Chamieh C, Liabeuf S, Massy Z. Uremic toxins and cardiovascular risk in chronic kidney disease: what have we learned recently beyond the past findings? Toxins. 2022;14:280. 35448889 10.3390/toxins14040280 PMC9028122

[22] Cohen G, Hörl WH. Immune dysfunction in uremia—an update. Toxins. 2012;4:962–90. 23202302 10.3390/toxins4110962 PMC3509694

[23] Zhu Z, Hu J, Chen Z, Feng J, Yang X, Liang W, et al. Transition of acute kidney injury to chronic kidney disease: role of metabolic reprogramming. Metabolism. 2022;131:155194. 10.1016/j.metabol.2022.155194. 35346693 10.1016/j.metabol.2022.155194

[24] Bai Z-h, Guo X-q. Dong R, Lei N, Pei Hh, Wang H: a nomogram to predict the 28-day mortality of critically ill patients with acute kidney injury and treated with continuous renal replacement therapy. Am J Med Sci. 2021;361:607–15. 10.1016/j.amjms.2020.11.028. 33288206 10.1016/j.amjms.2020.11.028

[25] Kuwatsuru Y, Hirano T, Wakabayashi R, Ishisaki JY, Sokooshi H, Kuwatsuru R. Changes in renal function over time in outpatients with eGFR ≥ 30 mL/min/1.73 m2: implication for timing of renal function testing before contrast-enhanced CT imaging. Jpn J Radiol. 2023;41:994–1006. 10.1007/s11604-023-01425-y. 37040025 10.1007/s11604-023-01425-y PMC10469099

[26] Wang J, Chen Y, Lu M, You D, Yang Z. A comparative study of three trajectory analysis methods. Chin J Health Stat. 2024;41:331–8.

