# Supplementary material for: Association of different serum creatinine trajectories with 28-day mortality in patients with acute kidney injury on chronic kidney disease: based on the MIMIC-IV database
Source: Eur J Med Res. 2025 Dec 15;31:101. doi: 10.1186/s40001-025-03632-x (PMC12821818; doi:10.1186/s40001-025-03632-x)
Supplement: Supplementary file 1 — Supplementary material 1. [file 40001_2025_3632_MOESM1_ESM.docx]

|  | level | Female | Male | *P* |
| --- | --- | --- | --- | --- |
| Trajectory group | G1 | 781 (27.1) | 981 (19.7) | <0.001 |
|  | G2 | 280 (9.7) | 635 (12.8) |  |
|  | G3 | 694 (24.1) | 1323 (26.6) |  |
|  | G4 | 600 (20.8) | 1107 (22.3) |  |
|  | G5 | 527 (18.3) | 924 (18.6) |  |

**Supplementary Table 1**: Sex Differences in Trajectory Groups
